# PTSD, dysregulation profile and substance use: exploring differences in a sample of adolescents in an outpatient clinic

**DOI:** 10.3389/frcha.2024.1421486

**Published:** 2024-12-19

**Authors:** Emely Reyentanz, Lukas A. Basedow, Veit Roessner, Yulia Golub

**Affiliations:** ^1^ Department of Child and Adolescent Psychiatry, Carl von Ossietzky Universität Oldenburg, Oldenburg, Germany; ^2^ Department of Child and Adolescent Psychiatry, Faculty of Medicine, Technische Universität Dresden, Dresden, Germany; ^3^ Fachbereich Psychologie, Klinische Psychologie und Psychotherapie, Phillips Universität Marburg, Marburg, Germany

**Keywords:** adolescents, traumatic events, PTSD–posttraumatic stress disorder, dysregulation profile, substance use

## Abstract

**Introduction:**

Experiencing traumatic events (TEs), especially interpersonal TEs, is related to an increased risk of developing post-traumatic stress disorder (PTSD). Both TEs and PTSD are associated with a higher risk of substance use and problems in emotion regulation. Little is known about the associations between specific types of TEs, problems with general self-regulation (including cognitive and behavioral components) and substance use severity in adolescents. Knowledge on these associations could provide important approaches for prevention and therapy for adolescents with a history of trauma.

**Methods:**

This study investigated associations between different types of TEs and PTSD, self-regulation and substance use severity. Moreover, participants were categorized into three groups according to their trauma status: (I) no history of TEs (noTEs), (II) history of TEs but no PTSD diagnosis (TEs), and (III) history of TEs and PTSD diagnosis (PTSD). Differences between the three groups were analyzed in terms of self-regulation and substance use severity. Our sample consisted of *N* = 89 adolescents aged 12 to 18 years in a child and adolescent psychiatric outpatient clinic in Germany. Substance use severity was only assessed in a smaller subsample (*n* = 37). Data were obtained from standardized diagnostic procedures and included information on types of TEs and PTSD diagnosis according to ICD-10, problems in self-regulation assessed with the Child Behavior Checklist (CBCL)/ Youth Self Report (YSR) Dysregulation Profile (DP), and substance use severity measured with the Drug Use Disorders Identification Test (DUDIT).

**Results:**

We found that interpersonal TEs were significantly associated with higher rates of PTSD diagnosis compared to non-interpersonal TEs. We found no significant associations between different types of TEs and both problems in self-regulation and substance use severity. Moreover, our findings do not indicate differences in both self-regulation and substance use severity between trauma statuses (noTEs, TEs, PTSD).

**Discussion:**

Future studies should consider other characteristics of TEs such as timing and duration when investigating associations with self-regulation. Longitudinal studies are needed to investigate developmental pathways, as a better understanding of the role of characteristics of TEs and self-regulation in the development of PTSD and substance use problems would provide opportunities for prevention and therapy for trauma-exposed patients.

## 1 Introduction

Throughout the lifespan, adolescence represents the period of highest risk for experiencing traumatic events (TEs) (1), with a correlation between a greater number of exposures to TEs over time and an increased likelihood of developing post-traumatic stress disorder (PTSD) (2). PTSD is defined as a specific reaction that may occur after the exposure to one or more TEs typically characterized by their “threatening or catastrophic nature”. It is manifested by the symptoms of re-experiencing in the form of flashbacks or nightmares, avoidance of trauma-associated stimuli and of heightened arousal (3).

Previous studies indicate a number of risk factors that influence the risk of developing PTSD following exposure to TEs. Certain sociodemographic risk factors such as female gender (4) or younger age in children (5) are associated with increased risk. Further findings suggest that both the quantity and severity of TEs may be a reliable predictor of the development of PTSD (6–8). TEs can be divided into interpersonal (“man-made” trauma, e.g., physical or sexual assault) and non-interpersonal (e.g., natural disasters, car accidents) types (9, 10). Different types of TEs have been demonstrated to influence the likelihood of developing PTSD. Research in adults indicates that interpersonal TEs are linked to a higher risk of developing PTSD compared to non-interpersonal TEs (11, 12). In particular, TEs related to violence, war (13, 14) and sexual assault are correlated with a higher risk of developing PTSD (7, 15). These associations have been investigated less frequently in adolescents. One study of children and adolescents aged 8–17 years aligns with findings from adult studies indicating that interpersonal TEs are associated with a higher risk of developing PTSD, while this is not the case for non-interpersonal TEs (16). In contrast, another study found no association between exposure to violence and the risk of PTSD in adolescents (17).

Both the experience of TEs and a PTSD have been found to be related to substance use (18, 19) and problems in emotion regulation (20, 21). Emotion regulation is a facet of self-regulation, a multidimensional construct that encompasses emotional, cognitive and behavioral regulation. It involves behaviors aimed at achieving goals or adapting to a context and is characterized by activation or inhibition (22–25). Self-regulation is thought to play a pivotal role in the development of psychiatric disorders across the lifespan (26–29).

The AAA-profile of the Child Behavior Checklist (CBCL) (30) comprises the scales *anxiety/depression*, *attention problems* and *aggressive behavior* and was initially regarded as a measure of juvenile bipolar disorder. Nowadays, it is thought to be a composite measure of emotional, cognitive and behavioral dysregulation (31) and is thus referred to as the Dysregulation Profile (DP). It can also be assessed using the corresponding self-report form of the CBCL, the Youth Self Report (YSR) (32, 33). Higher DP scores in YSR and CBCL indicate higher (emotional, cognitive and behavioral) dysregulation and thus more severe problems with self-regulation. Leibenluft, Charney (34) corroborated these findings and proposed the existence of a broad phenotype of severe mood and behavioral dysregulation characterized, for example, by increased reactivity and hyperarousal. They hypothesized that many individuals with at least one psychiatric diagnosis meet the criteria for this phenotype. Emotion dysregulation has been the subject of numerous studies investigating associations with TEs and PTSD. It has been shown to be influenced by the type of trauma, with higher emotional dysregulation being associated with interpersonal TEs compared to non-interpersonal TEs (35–37) and being a risk factor for the development of PTSD (36, 37). Moreover, emotion dysregulation appears to mediate the relationship between TEs and PTSD symptoms (37, 38). However, research on the relationship between self-regulation, encompassing cognitive and behavioral components, and both TEs and PTSD is lacking, although knowledge on these associations may provide further avenues for early prevention, for identifying high-risk groups prone to develop severe psychiatric problems such as PTSD, and for optimizing treatment of PTSD.

TEs and PTSD have also been linked to substance use disorder (SUD) in both adolescents and adults (18, 19). Basting, Medenblik (39) demonstrated in a sample of adults that a higher number of adverse childhood experiences is associated with the highest risk for posttraumatic stress symptoms and substance use problems. Several studies also indicate that adverse childhood experiences are associated with illicit substance use in adolescence (40, 41). Longitudinal studies confirm these results and indicate that adverse childhood experiences increase the risk for substance use in adolescence (42, 43). Some studies with adult samples have reported stronger associations between interpersonal TEs and later substance use (12, 44). One possible explanation for this could be that interpersonal TEs are usually related to more severe PTSD symptoms compared to non-interpersonal TEs (45). This in turn could be associated with greater substance use to cope with these symptoms (46, 47). On the other hand, reviews have not consistently confirmed these findings about the relationship for both adolescents and adults (48). In addition to examining the relationships between interpersonal and non-interpersonal TEs and substance use, no studies to date have examined the relationships between specific TEs and substance use among adolescents.

Previous studies also suggest associations between dysregulation and SUD. Clark, Donnellan (49) provide evidence that dysregulation, measured as temperamental reactivity, can predict later substance use in adolescence. Holtmann, Buchmann (50) showed that higher scores on the CBCL-DP at age 8 or 11 are associated with a higher risk of substance use at age 19. The authors discuss that dysregulation could follow different developmental trajectories. For example, SUD may reflect a type of (behavioral) dysregulation in adolescence.

To the best of our knowledge, this study is the first to investigate the relationship between specific types of TEs, dysregulation and SUD severity in a sample of adolescents. In addition, for the first time in this context, we will examine the DP as a more comprehensive measure of self-regulation, encompassing emotional, behavioral, and cognitive components. We will investigate its associations with substance use severity, as well as differences in DP and substance use severity in adolescents in three groups: (i) no history of TEs (noTEs), (ii) history of TEs but no PTSD (TEs), and (iii) history of TEs and PTSD (PTSD). While previous studies have focused on emotional regulation, this study is the first to examine a broader concept of self-regulation including emotional, behavioral and cognitive self-regulation in the context of trauma and substance use. This allows us to show for the first time whether there are abnormalities in self-regulation in patients in a psychiatric outpatient clinic. In addition, the current study's findings could provide important information for the optimization of prevention and therapy, as dysregulation could be specifically addressed during interventions. Of course, longitudinal studies would be necessary in the future to analyze causal relationships, but the results of the current study could be used to generate research questions for further investigation of the relationships and to investigate the role of emotional, behavioral and cognitive dysregulation in the development of psychopathology. Our study therefore has four objectives: Firstly, we seek to explore the relationship between different types of TEs and the risk of current PTSD in a sample of adolescents in two outpatient clinics. Building upon previous research, we expect trauma types to differ in their ability to explain PTSD and assume that experiencing interpersonal TEs such as war, terrorism and sexual assault will increase the risk for a current PTSD diagnosis compared to non-interpersonal TEs. Secondly, we will examine associations between different types of TEs, PTSD and DP as a measure of emotional, cognitive and behavioral dysregulation in adolescents. Previous findings on emotion dysregulation suggest the possibility of a stronger relationship between interpersonal TEs and dysregulation compared to non-interpersonal TEs. Moreover, we hypothesize that DP will be increased after TEs and in individuals diagnosed with PTSD compared to the noTEs group. Thirdly, we will investigate associations between TEs and substance use severity in adolescents. We assume that the association will be stronger for interpersonal TEs. In addition, we expect higher substance use severity in TEs and PTSD groups compared to noTEs group. Fourthly, the relationship between DP and substance use severity will be analyzed. We expect a positive correlation between DP and substance use severity.

In summary, a deeper understanding on the associations between TEs, PTSD, DP and substance use can provide important implication for prevention and therapy for children and adolescents, especially for those with a history of TEs.

## 2 Methods

### 2.1 Procedure

Data collection was embedded into the standard diagnostic procedure during admission to the Outpatient Clinic for Adolescent Substance Abuse and the Outpatient Clinic for Child and Adolescent Trauma, University Hospital C. G. Carus Dresden, Germany. After comprehensive verbal and written information, both the participants and their legal guardians gave their written informed consent for the anonymized diagnostic data to be used for research. All procedures were approved by the Institutional Review Board of the University Hospital C. G. Carus Dresden (EK 66022018). Patients did not receive reimbursements for participation in analyzed assessments.

### 2.2 Participants

Between November 2017 and January 2023, *N* = 740 patients contacted the outpatient clinics. *N* = 569 patients and their legal guardians agreed to the diagnostic data being used for research. We excluded patients who did not return CBCL and/or YSR questionnaires (*N* = 251), patients who were younger than 12 years of age (*N* = 80) as this study focuses on adolescents, patients with missing information on their age or gender (*N* = 4), patients with missing data on whether or not they had a history of TEs (*N* = 136), and patients with missing data on whether or not they had a PTSD diagnosis (*N* = 9) resulting in a final sample of *N* = 89 adolescents. Patients were 12.7–18.0 years old (*M* = 15.66, *SD* = 1.38). The sample consisted of *N* = 33 males (37.1%) and *N* = 56 females (62.9%). Participants were divided into three groups according to their trauma status: no history of TEs (noTEs), a history of TEs but no PTSD (TEs), and PTSD (PTSD).

### 2.3 Measures

#### 2.3.1 PTSD diagnosis

Diagnoses were made in the outpatient clinics according to ICD-10 criteria. The diagnoses relied on the combination of information from (1) multiple individual meetings between clinic staff, adolescents, and their caregivers, (2) the German version of the UCLA Child/Adolescent PTSD Reaction Index for DSM-5 (51) and (3) the Diagnostic Interview for psychiatric disorders in children and adolescents [Diagnostisches Interview bei psychischen Störungen im Kindes- und Jugendalter; J-DIPS] (52, 53).

#### 2.3.2 Dysregulation profile

A DP can be determined using the Youth Self Report (YSR/11–18) (54) and the Child Behaviour Checklist (CBCL/4–18) (30). The YSR, a self-report form for adolescents aged 11–18, and the corresponding caregiver report form, the CBCL/4–18, consist of 120 items which are answered on a 3-point-scale (0 = *not applicable*, 1 = *sometimes*, 2 = *frequently*). The DP is obtained by summing the t-values of the scales *anxiety/depression*, *attention problems* and *aggressive behavior*, which are assumed to reflect measures of emotional, cognitive and behavioral dysregulation, respectively (55, 56). This three-factor structure of the CBCL-DP was confirmed and validated in previous studies (57). It is therefore assumed that the DP reflects an overall measurement of dysregulation (55, 56). YSR/11–17 and CBCL/4–18 were handed out to patients and parents at the first consultation appointment. If both parents completed the CBCL/4–18, the mean of the two scores was calculated.

#### 2.3.3 Substance use severity

The Drug Use Disorders Identification Test (DUDIT) is a self-report screening questionnaire consisting of 11 items to assess problems related to illegal drug use (58). Items 1–9 are rated on a 5-point Likert scale and items 10 and 11 are rated on a 3-point Likert scale. A total score with a maximum of 44 can be calculated by summing all item scores (58). The questionnaire was developed to assess problematic use of illegal drugs and initially evaluated in a Swedish adult sample (58). It is available free of charge in several languages from the European Monitoring Centre for Drugs and Drug Addiction (59) and has proven to be a useful and reliable diagnostic tool for assessing drug use in adolescents (60).

#### 2.3.4 Control variables

Information on the age and gender of the adolescents was collected using a standardized generic questionnaire at the first consultation in the outpatient clinics.

#### 2.3.5 Types of traumatic events

The types of TEs were determined using information assessed by clinical staff during the diagnostic process and/or information from the UCLA Child/Adolescent PTSD Reaction Index for DSM-5 (61). The information on traumatic events (TEs) was categorized according to the types of TEs assessed in the UCLA Child/Adolescent PTSD Reaction Index for DSM-5 (Steinberg et al., 2004). These types of TEs are: natural disaster; serious accident; war terrorism; domestic violence; a family member experiencing physical assault; physical assault, shots or threats of serious injury; witnessing physical attacks, shootings or the death of others; saw dead body; sexual assault; violent death or serious injury of a loved one; painful or threatening medical treatment; child neglect; others. These were divided into “interpersonal” or “non-interpersonal” TEs, and the following are assumed to be interpersonal TEs: war terrorism; domestic violence; family member experiencing physical assault; physical assault, shots or threats of serious injury; witnessing physical attacks, shootings or the death of other; sexual assault; violent death or serious injury of a loved one; child neglect.

### 2.4 Statistical analysis

All analyses were conducted using IBM SPSS Statistics, version 29.0. To investigate the differences in the likelihood of a PTSD diagnosis between adolescents with and without interpersonal TEs, we performed a chi square test with the groups *TEs* and *PTSD* as well as *no interpersonal TEs* and *at least 1 interpersonal TE*. To examine how different types of TEs were related to the likelihood of a PTSD diagnosis, we conducted a binary logistic regression. The types of TEs were included as dichotomous variables (yes/no) indicating whether the event was experienced or not.

*T*-tests were conducted to test for differences in YSR-DP and CBCL-DP between patients with and without a history of interpersonal trauma. Both measures are used because previous studies indicate differences between self-reports and parental reports of psychopathology and recommend using both to obtain more valid information (62). Due to the violation of normal distributions, a non-parametric Mann–Whitney *U* test was also performed. To further investigate how different types of TEs are associated with YSR-DP and CBCL-DP linear regressions were conducted with the different types of TEs as independent variables and YSR-DP and CBCL-DP as dependent variables. To investigate whether there are differences in YSR-DP and CBCL-DP depending on trauma status, a Multivariate analysis of variances (MANCOVA) was performed with trauma status (noTE/TE/PTSD) as a group factor, age and gender as control variables and YSR-DP and CBCL-DP as dependent variables.

*T*-tests were conducted to examine differences in the DUDIT score between patients with and without a history of interpersonal trauma. To further investigate how the different types of TEs were related to the DUDIT score, we performed a linear regression with the different types of TEs as independent variables and the DUDIT score as the dependent variable. To investigate the differences in DUDIT score depending on trauma status, a one-way ANOVA was conducted with trauma status (noTE/TE/PTSD) as the group factor and DUDIT score as the dependent variable.

Due to missing data in DUDIT questionnaires, the sample size is smaller for analyses that include the DUDIT as an outcome measure. No adjustment for multiple testing was made in any of the analyses, as these analyses were intended to be explorative and hypothesis-generating to guide future work in this area. An alpha value of 0.05 was used as the threshold for statistical significance. The effect sizes were classified according to Cohen (63) into small effects (|*η*^2^| ≥ .01), medium effects (|*η*^2^| ≥ .06), and large effects (|*η*^2^| ≥ .14).

## 3 Results

### 3.1 Traumatic events and PTSD

A chi-square test investigating the association between interpersonal TEs and PTSD diagnosis indicates that adolescents with interpersonal TEs show higher rates of PTSD [*χ*²(1) = 6.78, *p* = .009, *φ* = 0.276] (see Table 1).

**Table 1:** Association between interpersonal TEs and PTSD diagnosis (*n* = 89).

	No Interpersonal TEs *N* (%)	At least 1 interpersonal TE *N* (%)	Total *N* (%)
No PTSD *N* (%)	26 (92.9%)	41 (67.2%)	67 (75.3%)
PTSD *N* (%)	2 (7.1%)	20 (32.8%)	22 (24.7%)
Total *N* (%)	28 (100%)	61 (100%)	89 (100%)

The binomial logistic regression model was statistically significant, *χ*²(15) = 30.71, *p* = .010, resulting in a medium amount of explained variance (Backhaus et al., 2003), as shown by Nagelkerke's *R*² = .466. The overall percentage of accuracy in classification was 83.8% with a sensitivity of 57.1% and a specificity of 93.2%. The variable sexual assault contributed significantly to the risk of PTSD (*p* = .035, OR = 6.595, 95% CI [21.138, 38.212]. All model coefficients and odds can be found in Table 2.

**Table 2:** Binary logistical regression with age, gender and types of TEs as predictors (*n* = 89).

Traumatic event	*N* (with PTSD)	Unstandardized regression coefficient *B*	Standardized regression coefficient *β*	*p*	95% CI of *B*
Age		.365	1.441	.176	[.848; 2.446]
Gender		1.868	6.475	.072	[.847; 49.489]
Natural disaster	7 (1)	.447	1.564	.791	[.057; 42.895]
Serious accident	3 (0)	−21.599	.000	.999	[.000;.]
War/terrorism	3 (2)	3.192	24.337	.055	[.933; 634.622]
Domestic violence	11 (2)	4.084	59.383	.052	[.973; 3,625.908]
Family member experiencing physical assault	10 (2)	−.919	.399	.612	[.011; 13.903]
Physical assault, shots or threats of serious injury	19 (6)	1.269	3.556	.143	[.651; 19.425]
Witnessing physical attacks, shootings or the Death of others	13 (3)	1.440	4.219	.278	[.313; 56.957]
Saw dead body	5 (1)	.684	1.981	.798	[.011; 370.979]
Sexual assault	28 (12)	1.886	6.595	.035	[1.138; 38.212]
Violent death or serious injury of a loved one	12 (1)	−23.168	.000	.998	[.000;.]
Painful or threatening medical treatment	5 (0)	−20.511	.000	.999	[.000;.]
Child neglect	6 (1)	.226	1.254	.912	[.022; 70.340]
Others	21 (5)	.906	2.473	.413	[.283; 21.601]

### 3.2 Traumatic events, PTSD and dysregulation profile

#### 3.2.1 Associations between types of traumatic events and dysregulation profile

*T*-tests to test for differences in YSR-DP and CBCL-DP between patients with and without a history of interpersonal TEs obtained no significant differences between groups for YSR-DP [*t*(87) = 1.33, *p* = .187; *U* = 689.50, *p* = .146] and for CBCL-DP [*t*(87) = −0.409, *p* = .683].

Linear regressions were conducted to test whether there were significant associations between age, gender, and types of TEs and both YSR-DP and CBCL-DP. All model coefficients can be found in Table 3. The types of TEs were not able to explain YSR-DP [*F*(15, 64) = 1.034, *p* = .434] and CBCL-DP [*F*(15, 64) = 0.756, *p* = .719].

**Table 3:** Model coefficients of linear regression analyses on YSR-DP, CBCL-DP and DUDIT*.*

	YSR-DP	CBCL-DP	DUDIT
	Unstandardized regression Coefficient *B* (*p*) [95% CI of *B*] Standardized Regression Coefficient Beta		
Age	−0.84 (*p* = .66)	−0.83 (*p* = .691)	3.20 (*p* = .097)
	[−4.62; 2.94]	[−4.98; 3.32]	[−0.66; 7.06]
	*β* = −0.06	*β* = −0.05	*β* = .449
Gender	13.85 (*p* = .025)	7.61 (*p* = .256)	6.43 (*p* = .148)
	[1.78; 25.91]	[−5.66; 20.87]	[−2.58; 15.43]
	*β* = 0.31	*β* = 0.16	*β* = .37
Natural disaster	−18.13 (*p* = .11)	−10.81 (*p* = .383)	−4.93 (*p* = .373)
	[−40.51; 4.25]	[−35.43; 13.80]	[−16.41; 6.56]
	*β* = −0.21	*β* = −0.12	*β* = −.20
Serious accident	10.84 (*p* = .510)	−10.86 (*p* = .548)	–
	[−21.84; 43.52]	[−46.79; 25.08]	
	*β* = 0.08	*β* = −0.08	
War terrorism	3.13 (*p* = .818)	12.97 (*p* = .387)	–
	[−23.94; 30.20]	[−16.79; 42.74]	
	*β* = 0.03	*β* = 0.11	
Domestic violence	−23.06 (*p* = .057)	−14.98 (*p* = .256)	−6.17 (*p* = .572)
	[−46.80; 0.68]	[−41.09; 11.12]	[−29.06; 16.72]
	*β* = −0.36	*β* = −0.22	*β* = −.270
Family member experiencing physical assault	11.17 (*p* = .330)	13.58 (*p* = .282)	8.72 (*p* = .236)
	[−11.58; 33.92]	[−11.44; 38.59]	[−6.37; 23.81]
	*β* = 0.16	*β* = 0.18	*β* = .35
Physical assault, shots or threats of serious injury	−1.78 (*p* = .774)	−4.66 (*p* = .497)	2.90 (*p* = .563)
	[−14.16; 10.60]	[−18.27; 8.96]	[−7.61; 13.42]
	*β* = −0.04	*β* = −0.09	*β* = .14
Witnessing physical attacks, shootings or the death of others	4.47 (*p* = .609)	−1.92 (*p* = .841)	1.97 (*p* = .696)
	[−12.88; 21.82]	[−21.00; 17.16]	[−8.62; 12.55]
	*β* = 0.08	*β* = −0.03	*β* = .11
Saw dead body	26.49 (*p* = .060)	8.04 (*p* = .600)	10.66 (*p* = .200)
	[−1.18; 54.16]	[−22.39; 38.47]	[−6.34; 27.65]
	*β* = 0.27	*β* = 0.08	*β* = .38
Sexual assault	−8.46 (*p* = .160)	−5.41 (*p* = .412)	−6.46 (*p* = .324)
	[−20.35; 3.43]	[−18.48; 7.67]	[−20.04; 7.11]
	*β* = −0.19	*β* = −0.11	*β* = −.28
Violent death or serious injury of a loved one	3.32 (*p* = .738)	1.19 (*p* = .913)	−3.11 (*p* = .648)
	[−16.42; 23.05]	[−20.51; 22.89]	[−17.37; 11.16]
	*β* = 0.05	*β* = 0.02	*β* = −.15
Painful or threatening medical treatment	−13.16 (*p* = .348)	−3.88 (*p* = .801)	−3.58 (*p* = .635)
	[−40.96; 14.64]	[−34.45; 26.69]	[−19.44; 12.27]
	*β* = −0.15	*β* = −0.04	*β* = −.16
Child neglect	8.34 (*p* = .493)	29.15 (*p* = .032)	−5.05 (*p* = .68)
	[−15.82; 32.49]	[2.59; 55.72]	[−30.70; 20.60]
	*β* = 0.10	*β* = 0.31	*β* = −.18
Other	−11.25 (*p* = .110)	−10.30 (*p* = .182)	2.16 (*p* = .737)
	[−25.11; 2.61]	[−25.54; 4.94]	[−11.41; 15.74]
	*β* = −0.23	*β* = −0.19	*β* = .10
Test variables			
Corrected *R*^2^	*R*^2^ = .195	*R*^2^ = .151	*R*^2^ = .525
*F* (*p*-value)	*F*(15, 64) = 1.034 (*p* = .434)	*F*(15, 64) = 0.756 (*p* = .719)	*F*(13, 14) = 1.190 (*p* = .375)

#### 3.2.2 Group differences between noTEs, TEs and PTSD in dysregulation profile

A MANCOVA to investigate whether there are differences in DP depending on trauma status revealed no significant differences between groups [*F*(4, 168) = 1.543, *p* = .192, *η*²_part_ = .035, Wilk's Λ = .930] (see also Table 4).

**Table 4:** Group differences in YSR-DP and CBCL-DP (*n* = 89) and DUDIT (*n* = 37).

	YSR-DP	CBCL-DP	DUDIT
noTE	*n* = 14	*n* = 14	*n* = 8
*M* (*SD*)	193.86 (20.78)	189.75 (23.67)	13.13 (12.26)
TE	*n* = 53	*n* = 53	*n* = 27
*M* (*SD*)	182.92 (20.90)	191.08 (23.92)	14.78 (9.01)
PTSD	*n* = 22	*n* = 22	*n* = 2
*M* (*SD*)	184.91 (22.49)	184.09 (20.91)	14.50 (4.95)
noTEs vs. TEs vs. PTSD	*F*(2, 84) = 1.531; *p* = .222; *η*^2^_part_ = .035	*F*(2, 84) = 1.022; *p* = .364; *η*^2^_part_ = .024	*F*(2, 34) = 0.090, *p* = .914, *η*² = .005

### 3.3 Traumatic events, PTSD and substance use severity

#### 3.3.1 Associations between types of traumatic events and substance use severity

*T*-tests investigating differences in DUDIT score between patients with (*N* = 23, *M_DUDIT_* = 15.00, *SD* = 9.43) and without a history of interpersonal TEs (*N* = 14, *M_DUDIT_* = 13.43, *SD* = 9.71) revealed no significant difference in DUDIT score between groups [*t*(35) = −.762, *p* = .630].

A linear regression testing for significant associations between types of TEs and DUDIT score showed that types of TEs were not able to explain DUDIT scores [*F*(13, 14) = 1.190, *p* = .375]. All model coefficients can be found in Table 3.

#### 3.3.2 Group differences between noTEs, TEs and PTSD in substance use severity

A one-way ANOVA to investigate differences in DUDIT score depending on trauma status did not reveal any statistically significant difference between groups [*F*(2, 34) = 0.090, *p* = .914, *η*² = .005] (see also Table 4).

### 3.4 Dysregulation and substance use severity

The Pearson correlation revealed no significant correlations between DUDIT score and YSR-DP (*r* = .275, *p* = .099) and between DUDIT score and CBCL-DP (*r* = .128, *p* = .451).

## 4 Discussion

In this study, we investigated a sample of adolescents in two German child psychiatric outpatient clinics. Our first aim was to examine relationships between different types of TEs and PTSD diagnoses. Secondly, we aimed to investigate the relationship between different types of TEs and DP and differences in DP according to trauma status (noTEs, TEs, PTSD). Third, we wanted to examine the relationship between types of TEs and substance use severity as well as differences between the three groups (noTEs, TEs, PTSD) in terms of substance use severity. Fourth, we aimed to investigate the association between DP and substance use severity. Our findings suggest that adolescents who have experienced interpersonal TEs exhibit higher rates of PTSD compared to adolescents who have not experienced interpersonal TEs. In particular, the experience of sexual assault contributed significantly to the explanation of PTSD. We found no associations between the different types of TEs and both DP and substance use severity and no differences between the three groups (noTEs, TEs, PTSD) in terms of DP and substance use severity. Moreover, substance use severity did not correlate significantly with both YSR-DP and CBCL-DP.

In line with previous findings, this study showed a stronger association between interpersonal TEs and PTSD compared to non-interpersonal TEs (9, 11, 12). In particular, the experience of sexual assault increased the risk of PTSD in our study. These results should be further investigated in longitudinal studies as they could provide important information for specific preventive measures against the development of PTSD for those affected by this type of TEs. In this study we found no association between the different types of TEs and both self- and parent-reported DP. In addition, no differences in DP were found between the three groups (noTEs, TEs, PTSD) in this study. As we know from previous research, the association between TEs and PTSD is affected by several factors (64, 65). For example, a current meta-analysis investigated peritraumatic risk factors for PTSD in children and adolescents (64). The authors reported that subjective threat during the trauma appears to increase the likelihood of developing PTSD in children and adolescents, although this factor alone cannot explain it. Nevertheless, these results suggest that, in addition to objective variables such as psychiatric diagnoses, the subjective experience of TEs appears to be related to posttraumatic consequences and could therefore also be associated with DP (64).

Several previous studies have linked emotion regulation to PTSD symptom severity (66), whereas we only compared DP between groups with and without PTSD diagnosis. It could also be that PTSD diagnoses are not generally characterized by higher DP scores, but that the severity of PTSD symptoms correlates with DP. In this context, it should also be noted that arousal or irritability are also part of the diagnostic criteria of PTSD (3). In our study, it is not possible to distinguish between dysregulation as a personality trait, dysregulation as a reaction following TEs and potential risk factor for developing PTSD and dysregulation as a PTSD symptom.

Furthermore, it has been observed that dysregulation is associated with psychopathology in general (67, 68). For example, Dölitzsch et al. (69), investigated children and adolescents aged 10–18 years in residential care. They compared those with T-scores ≥67 on the AAA-scales of YSR/CBCL with those below this cutoff and found that patients with a YSR-DP T-score ≥67 on the AAA-scales had more different psychiatric diagnoses than patients with a lower YSR-DP. These results suggest that the number of psychiatric diagnoses appears to be related to DP and should therefore be considered in future studies investigating relationships between TEs, PTSD and DP.

Due to the cross-sectional design of our study, we were not able to investigate causal relationships and developmental trajectories of DP and the development of PTSD. As Deutz et al. (70) show, YSR-DP and CBCL-DP seem to follow a non-linear developmental trajectory that appears to be relatively stable from 4 to 17 years, with a peak in adolescence. These findings suggest that early development and TEs in early childhood may have a greater impact on dysregulation, whereas the experience of TEs later in life may play a subordinate role. Therefore, it would be interesting for future research to consider not only the type of TEs but also the timing of TEs when investigating associations with dysregulation. Of course, additional factors that contribute to dysregulation independently of the TEs should also be included in the investigations.

Due to the design of our study, we were only able to investigate cross-sectional associations. Future studies should focus on longitudinal investigations to investigate causal relationships between dysregulation, TEs and PTSD as well as possible factors influencing the associations. These investigations would be an important step towards a better understanding of the developmental pathways of dysregulation and PTSD and would provide important approaches for prevention and intervention options for children and adolescents with a history of TEs.

We found no significant differences in substance use severity between adolescents with and without a history of interpersonal TEs. In accordance with previous studies, our results reveal that patients with a history of interpersonal trauma had, on average, higher mean substance use severity than patients without a history of interpersonal trauma (12, 44).

Besides, we found no significant associations between the different types of TEs and substance use severity. One explanation is that, in addition to the type of TEs, other factors could also mediate the relationship between TEs and substance use severity. Khoury et al. (71), investigated associations between physical, sexual and emotional abuse in childhood and the use of alcohol, cocaine, opiates, cannabis and tobacco in adulthood. They reported associations between certain types of TEs and the use of different substances. Because our sample was too small to conduct analyses for different subsamples of substances, we were not able to investigate these associations.

Previous research also suggests that the association between TEs and substance use severity is mediated by trauma-related factors such as posttraumatic stress symptoms (19, 72). As posited by the self-medication hypothesis, substance use serves as a coping mechanism for managing symptoms of PTSD (46, 47). Therefore, the use of certain substances appears to differ depending on trauma-related symptoms and the specific effects of the different substances (47, 73). For example, Dworkin et al. (74) reported an association between hyperarousal symptoms and cocaine use disorder, while others did not find this relationship (75, 76). Even less is known about these associations in adolescents. One study that examined associations between trauma symptom clusters and MDMA use found a relationship between avoidance symptoms and use of MDMA (77).

Overall, further research is needed to gain a better understanding of the association between types of TEs and substance use severity and potential mediating factors. A recent review suggests that early risks such as child maltreatment increase the risks for earlier and more severe substance use in adolescence and for later SUD (78), which emphasizes the importance of considering these associations in therapeutic approaches for at-risk adolescents.

In our study, we also found no significant differences in substance use severity between the three groups (noTEs, TEs, PTSD). It should be noted that only *n* = 2 adolescents were included in the PTSD group. However, the mean substance use severity scores were higher for the TEs and the PTSD groups compared to the noTEs group, although the differences were not significant. These findings are consistent with previous studies (18), although surprisingly the mean substance use severity was higher in the TEs group than in the PTSD group. Due to the small sample sizes results should be interpreted with caution.

Our results reveal no correlation between substance use severity and self-and parent-reported DP (79, 80). These results are in contrast to former research findings (49). It might be possible, that substance use is rather associated with emotion regulation than with general self-regulation in adolescents. In addition, previous studies indicate that the relationship between emotional dysregulation and substance use varies depending on the substance consumed (81). Characteristics such as substances used, but also frequency or amount could be taken into account in future studies.

Cooper et al. (82) made one of the first attempts to investigate the relationship between the three variables TEs, dysregulation and SUD in a sample of adolescents. Childhood maltreatment, substance use and emotional, behavioral and cognitive dysregulation were assessed at the age of 10–12 years and again 1, 2 and 3 years later. The results showed positive associations between the extent of childhood maltreatment and emotional and behavioral dysregulation as well as between emotional and behavioral dysregulation and substance use. Further, emotional and behavioral dysregulation mediated the association between childhood maltreatment and substance use. Although the findings of Cooper et al. (82) suggest a mediating role of dysregulation in the association between TEs and substance use, the assumption of linear relationships between predictor (TEs), mediator (dysregulation) and criterion (substance use) could not be confirmed in our study, so we were unable to investigate these associations in our sample. However, further investigations on this association would be important as they may provide important insights into the apparently complex developmental pathways and interactions of dysregulation and substance use severity in adolescents with a history of trauma.

As the present study is an exploratory study, no adjustment for multiple testing was applied. The results must therefore be interpreted cautiously. Additionally, the subsamples in the various analyses are limited and the analyses on substance use severity were only conducted with a smaller subsample due to the lack of DUDIT questionnaires. Since we used cross-sectional data, no conclusions about causal relationships can be drawn. Prospective longitudinal studies are needed to investigate causal relationships and developmental pathways of PTSD, dysregulation and substance use severity in adolescents with a history of TEs.

Altogether, our results suggest that interpersonal TEs are associated with a higher risk of PTSD, whereas we found no association between TEs and both DP and substance use severity in a sample of adolescents with trauma history in an outpatient clinic. Our findings underscore the significance of early interventions aimed at adolescents who have encountered interpersonal TEs. Longitudinal investigations are essential for elucidating causal relationships and developmental trajectories, while also considering comorbidities and attributes of traumatic events, including number, timing and duration. Furthermore, dysregulation should not only be investigated as a consequence or correlate of PTSD and substance use, but also as a risk factor, that develops as a function of various factors and could influence the development of psychopathology, especially after TEs. Longitudinal studies and comparisons between clinical and non-clinical samples could improve the understanding of the role of self-regulation in the development of psychiatric disorders. A deeper comprehension of these associations could provide valuable perspectives for prevention and therapeutic interventions for individuals who have been exposed to trauma.

## Data Availability

The raw data supporting the conclusions of this article will be made available by the authors, without undue reservation.
